# Author reply to what are the clinical signs of thiamine deficiency in elderly patients?

**DOI:** 10.1002/jgf2.484

**Published:** 2021-07-27

**Authors:** Ryuichi Ohta, Yoshinori Ryu, Shuzo Hattori, Chiaki Sano

**Affiliations:** ^1^ Community Care Unnan City Hospital Unnan Shimane Prefecture Japan; ^2^ Internal Medicine Unnan City Hospital Unnan Shimane Prefecture Japan; ^3^ Department of Community Medicine Management Faculty of Medicine Shimane University Izumo Shimane Prefecture Japan

To the Editor,

Onishi et al. responded productively to our research by adding the importance of the relationship between symptoms and vitamin B1 deficiency.^1^, ^2^ Vitamin B1 deficiency can be prevalent, especially among older people with critical conditions such as cancer, as suggested by the authors. As the population of the society ages, issues regarding vitamin B1 deficiency will become vital for the sustainability of communities. When it comes to considering the symptoms caused by vitamin B1 deficiency, the vagueness of the symptoms, background of patients, and clinical settings should be considered to clarify the relationship between symptoms and vitamin B1 deficiency.

The vagueness of the symptoms regarding vitamin B1 deficiency can make the diagnosis of vitamin B1 deficiency challenging. As the authors of the letter suggested, Wernicke encephalopathy can be critical because of high mortality and is diagnosed on the basis of typical symptoms, blood tests, and magnetic resonance imaging.^3^ However, vitamin B1 deficiency can produce various low‐yield symptoms such as fatigue, irritation, poor memory, and anorexia at an early stage.^4^ Clinicians should suspect the presence of vitamin B1 deficiency with low‐yield symptoms in case of the absence of typical symptoms of other diseases. In particular, general and family physicians have to deal with low‐yield symptoms more frequently as a gate opener than other specialists. Therefore, our research used clinical data from a rural community hospital where family and general internal medicine physicians suspected the presence of vitamin B1 deficiency in patients with various symptoms.

The perception and degree of symptoms can be dependent on patients’ background, clinical settings, and their help‐seeking behavior (HSB), which can make it difficult to investigate the relationship between symptoms and vitamin deficiencies. Older patients tend to experience symptoms in varying degrees because aging impinges on the sensitivity of perceiving symptoms.^5^ Chronic diseases such as diabetes, neuropathy, and atherosclerosis can inhibit them from clearly experiencing symptoms.^6^ In addition, mental conditions such as depression, panic disorders, and schizophrenia can change the perception of symptoms.^7^ Clinical settings such as outpatient and inpatient departments can affect patients’ mental condition.^5^ Furthermore, the symptoms can be modified by their HSB. Our previous research suggested the presence of various HSBs and the hesitance of their HSB with regard to medical professionals in rural contexts.^8^, ^9^ Therefore, rural people tried to medicate themselves by taking home remedies and over‐the‐counter drugs, which can change their symptoms. A comprehensive investigation of the relationship between symptoms and vitamin B1 deficiency should be performed with regard to various contexts and patient backgrounds.

I appreciate the letter of the authors regarding the present challenges for the clarification of the relationship between symptoms and vitamin deficiencies. In the present era, aging societies can lead to the exacerbation of the prevalence of vitamin B1 deficiencies, which may drive the progression of dementia and frailty in future. Future studies should investigate the prevalence of vitamin B1 deficiency in various contexts and improve older people's HSB for improvements in their quality of life.

## CONFLICT OF INTEREST

The authors declare no conflicts of interest with regard to this article.

## References

[1] Onishi H , Ishida M . What are the clinical signs of thiamine deficiency in elderly patients? J Gen Fam Med. 2021. 10.1002/jgf2.479 in press. PMC8721321 35004119

[2] Ohta R , Ryu Y , Hattori S . Association between transient appetite loss and vitamin B1 deficiency in elderly patients with suspected deficiency. J Gen Fam Med. 2020;22(3):128–33. 33977009 10.1002/jgf2.404 PMC8090839

[3] Ota Y , Capizzano AA , Moritani T , Naganawa S , Kurokawa R , Srinivasan A . Comprehensive review of Wernicke encephalopathy: pathophysiology, clinical symptoms and imaging findings. Jpn J Radiol. 2020;38(9):809–20. 32390125 10.1007/s11604-020-00989-3

[4] Fattal‐Valevski A . Thiamine (Vitamin B1). Evid Based Complement Alternat Med. 2011;16(1):12–20.

[5] Sirri L , Fava GA , Sonino N . The unifying concept of illness behavior. Psychother Psychosom. 2013;82(2):74–81. 23295460 10.1159/000343508

[6] Alberts JF , Sanderman R , Gerstenbluth I , van den Heuvel WJ . Sociocultural variations in help‐seeking behavior for everyday symptoms and chronic disorders. Health Policy. 1998;44(1):57–72. 10180202 10.1016/s0168-8510(98)00015-3

[7] Magaard JL , Seeralan T , Schulz H , Brütt AL . Factors associated with help‐seeking behaviour among individuals with major depression: A systematic review. PLoS One. 2017;12(5):e0176730. 28493904 10.1371/journal.pone.0176730 PMC5426609

[8] Ohta R , Ryu Y , Kitayuguchi J , Sano C , Könings KD . Educational intervention to improve citizen's healthcare participation perception in rural Japanese communities: a pilot study. Int J Environ Res Public Health. 2021;18(4):1782. 33673096 10.3390/ijerph18041782 PMC7918205

[9] Ohta R , Sato M , Ryu Y , Kitayuguchi J , Maeno T , Sano C . What resources do elderly people choose for managing their symptoms? Clarification of rural older people's choices of help‐seeking behaviors in Japan. BMC Health Serv Res. 2021;21(1):640. 34217269 10.1186/s12913-021-06684-x PMC8254357

